# Endoplasmic Reticulum Glycoprotein Quality Control Regulates CD1d Assembly and CD1d-mediated Antigen Presentation*[Fn FN2]

**DOI:** 10.1074/jbc.M113.474221

**Published:** 2013-04-24

**Authors:** Amit Kunte, Wei Zhang, Crina Paduraru, Natacha Veerapen, Liam R. Cox, Gurdyal S. Besra, Peter Cresswell

**Affiliations:** From the ‡ Section of Infectious Diseases, Department of Internal Medicine, and; § Department of Immunobiology, Howard Hughes Medical Institute, Yale University School of Medicine, New Haven, Connecticut 06520-8011 and; the ¶ School of Biosciences and; ‖ School of Chemistry, University of Birmingham, Edgbaston, Birmingham B15 2TT, United Kingdom

**Keywords:** Antigen Processing, ER Quality Control, Major Histocompatibility Complex (MHC), T cell, Transport

## Abstract

The non-classical major histocompatibility complex (MHC) homologue CD1d presents lipid antigens to innate-like lymphocytes called natural-killer T (NKT) cells. These cells, by virtue of their broad cytokine repertoire, shape innate and adaptive immune responses. Here, we have assessed the role of endoplasmic reticulum glycoprotein quality control in CD1d assembly and function, specifically the role of a key component of the quality control machinery, the enzyme UDP glucose glycoprotein glucosyltransferase (UGT1). We observe that in UGT1-deficient cells, CD1d associates prematurely with β_2_-microglobulin (β_2_m) and is able to rapidly exit the endoplasmic reticulum. At least some of these CD1d-β_2_m heterodimers are shorter-lived and can be rescued by provision of a defined exogenous antigen, α-galactosylceramide. Importantly, we show that in UGT1-deficient cells the CD1d-β_2_m heterodimers have altered antigenicity despite the fact that their cell surface levels are unchanged. We propose that UGT1 serves as a quality control checkpoint during CD1d assembly and further suggest that UGT1-mediated quality control can shape the lipid repertoire of newly synthesized CD1d. The quality control process may play a role in ensuring stability of exported CD1d-β_2_m complexes, in facilitating presentation of low abundance high affinity antigens, or in preventing deleterious responses to self lipids.

## Teaser

**Background:** Various lipid antigens presented by the glycoprotein CD1d strongly influence immune responses.

**Results:** In the absence of endoplasmic reticulum (ER) glycoprotein quality control, CD1d complexes are formed prematurely and have altered antigenicity.

**Conclusion:** ER glycoprotein quality control monitors CD1d assembly and likely influences the loaded lipid repertoire.

**Significance:** Our findings reveal a new mechanism for regulating CD1d-mediated antigen presentation.

## Introduction

The CD1 family of antigen-presenting molecules has been studied extensively and with increasing interest over the last several years. These non-polymorphic major histocompatibility complex (MHC) class I-like proteins are distinguished by their ability to present lipid antigens as opposed to peptides. CD1d, the sole member of the Group 2 CD1 molecules (1, 2), has received attention because of its ability to stimulate an innate-like subset of lymphocytes termed natural-killer T (NKT)^4^ cells. The most studied NKT cells (called invariant NKT (iNKT) cells) have T-cell receptors (TCRs) with limited diversity (3–5). This property, along with the ability of CD1d to bind and present a wide variety of lipids, allows for polyclonal activation in response to both exogenous and endogenous lipids. iNKT cells also have a memory-like phenotype and can respond to antigens in a matter of hours without the need for sensitization. This rapid response coupled with the ability to secrete copious amounts of cytokines enables iNKT cells to exert a major influence on ensuing immune responses. iNKT cells have been implicated in protective immunity to a large number of infections (6, 7) and in the pathogenesis of various inflammatory conditions (4). Similarly, iNKT cells have been implicated in tumor immunity and have potential as mediators of tumor immunotherapy (8).

There is significant variation in the nature and amplitude of the iNKT cell response elicited by various CD1d-presented lipids (for review, see Ref. 9). There has been great interest in uncovering the basis for these differences, and the mechanisms are manifold. Many reports have focused on the molecular underpinnings of CD1d-lipid-TCR interactions and their effect on NKT cell activation (for review, see Refs. 10 and 11). Structural studies have revealed that CD1d has an MHC-like antigen binding groove with two hydrophobic pockets. CD1d antigens, although varied, are all amphipathic. The hydrophobic portion (usually consisting of two aliphatic carbon chains) is buried in the two hydrophobic channels, and the polar head group (either sugar- or phosphate-based) is solvent-exposed. Because of this orientation, most of the actual contacts at the CD1d-TCR interface are mediated by the lipid head group and the CD1d α1 and α2 chains themselves. However, differences in the buried hydrophobic portions of the lipid clearly affect the function of the CD1d-lipid complex (12). This may result from altered lipid loading kinetics (12, 13) as well as from transmitted structural changes in the solvent-exposed surface of the CD1d protein (14). Therefore, multiple characteristics of the CD1d-lipid complex determine the specificity of iNKT cell responses. There are also other contributory factors, such as the site of loading on the antigen presenting cell (APC) (13), costimulatory properties of the APC (15), and the presence of cytokines (for review, see Ref. 7).

Similar to MHC class I, CD1d consists of a single-pass transmembrane polypeptide (the free heavy chain) complexed to soluble β_2_-microglobulin (β_2_m). After synthesis and translocation into the endoplasmic reticulum (ER), newly synthesized CD1d heavy chains are stabilized by interaction with the ER lectin chaperones calnexin and calreticulin (CRT) (16). Chaperone binding recruits ERp57, a thiol oxidoreductase that facilitates disulfide bond maturation. In contrast to MHC class I, β_2_m binding to CD1d occurs after heavy chain dissociation from the lectin chaperones. CD1d may also bind another ER-localized molecule, the lipid transfer protein microsomal triglyceride transfer protein (MTP) (17), which facilitates the acquisition of lipids by CD1d (18, 19). The assembled CD1d-β_2_m complexes, presumably containing ER-derived lipids (20, 21), continue on through the secretory pathway to the plasma membrane. Cell surface CD1d can be internalized via clathrin-mediated endocytosis and recycles multiple times through the endocytic pathway. A number of accessory molecules facilitate lipid exchange in the endocytic pathway, and this is believed to be a major site of antigen acquisition (for review, see Refs. 1 and 22).

Given that the structure of the CD1d-lipid complex is a key determinant of downstream NKT cell responses, we have been interested in exploring cellular quality control mechanisms that regulate the assembly of these complexes. A related question has been whether there is selectivity in the lipids loaded onto CD1d during assembly. Specific ER-loaded lipids may affect CD1d function by influencing the structure of the CD1d-β_2_m complex, by being directly sensed by iNKT cells (23), or by influencing the efficiency with which antigenic lipids can be exchanged for ER-derived lipids in post-ER compartments. The profile of lipids eluted from an ER-retained CD1d variant appears to reflect the composition of bulk ER lipids (21), but it is unclear whether this is a good representation of dynamic events in the synthesis and assembly of new complexes. Interestingly, in addition to facilitating folding, CRT binding also controls the rate of export of CD1d from the ER (24). In the absence of CRT, functional (and therefore presumably folded) CD1d-β_2_m complexes form and exit the ER at an accelerated rate. This suggests that CRT has a role in the active retention of folded heavy chains. Therefore, we hypothesized that CRT and the UGT1-mediated ER glycoprotein quality control cycle involving CRT may have a critical functional role in CD1d assembly.

UGT1-mediated glycoprotein quality control functions by modification of *N*-linked glycans on nascent glycoproteins (for review, see Refs. 25 and 26)). Newly synthesized glycoproteins are co-translationally modified with Glc_3_Man_9_GlcNAc_2_ glycans. These glycans are trimmed to a monoglucosylated form by the sequential action of glucosidase I and glucosidase II. These monoglucosylated glycans mediate interaction of the nascent glycoprotein with the lectin chaperones calnexin and CRT, which facilitate folding and disulfide bond maturation by recruiting the thiol oxidoreductase ERp57. Trimming of the final glucose by glucosidase II releases the glycoprotein from the chaperones. After further glycan processing, properly folded proteins are exported from the ER whereas terminally misfolded proteins are targeted for ER-associated degradation. Substrates that are near-native but not completely folded can enter a rescue pathway mediated by UGT1. The enzyme appears to preferentially recognize such substrates, possibly by sensing surface-exposed hydrophobic patches proximal to *N*-linked glycosylation sites in near-native molten globule like conformations, and reglucosylates the Man_9_GlcNAc_2_ glycan (27–29). This allows reentry of the glycoprotein into the calnexin/CRT-mediated folding cycle (30). We have recently shown that UGT1 preferentially reglucosylates MHC Class I molecules loaded with suboptimal peptides and is thereby able to rescue unstable, suboptimally loaded MHC Class I molecules from degradation (31, 32).

In this study we have used UGT1-deficient cells to assess the role of glycoprotein quality control in CD1d assembly and function. The results demonstrate that UGT1-mediated quality control regulates the assembly of CD1d-β_2_m complexes. In the absence of UGT1, we observe premature release of shorter-lived, potentially unstable complexes. Importantly, we were able to demonstrate that the quality control process affects the antigen presentation function of CD1d, possibly by affecting the repertoire of CD1d-bound lipids.

## EXPERIMENTAL PROCEDURES

### 

#### 

##### Cell Line, Plasmids, and Stable Transfectants

UGT1-deficient mouse embryonic fibroblasts, and their cognate WT cells were gifts from Maurizio Molinari (Institute for Research in Biomedicine, Bellinzona, Switzerland) (33). The fibroblasts and their transfectants were maintained in Iscove's modified Dulbecco's medium (Sigma) supplemented with 10% fetal bovine serum (Hyclone) at 37 °C in 5% CO_2_. Hybridomas N37-1H5a, N38-2C12, and N57-2C12 were gifts from Sebastian Joyce (Vanderbilt University, Nashville, TN) and have been described previously (34, 35). The DN32.D3 hybridoma was kindly provided by Dr. Albert Bendelac (University of Chicago) (36). The MSCV-UGT1-IRES-Thy1.1 (32) and MSCV-CD1d-IRES-GFP (24) constructs and their use to transduce mouse embryonic fibroblasts have been described previously

##### Antibodies and Reagents

The monoclonal mouse antibodies to human CD1d- CD1d51 and D5 have been previously described (37). Other antibodies used were biotinylated goat anti-green fluorescent protein (GFP) antibody (Rockland Immunochemicals) and rabbit anti-UGT1 (VAP-PT068; Stressgen). Endoglycosidase H (EndoH) and peptide *N*-glycanase F were obtained from New England Biolabs. Chloroquine was from Sigma. IL-2 was measured using an IL-2 enzyme-linked immunosorbent assay (ELISA) kit (BD Biosciences). α-Galactosylceramide (αGC) and α-galactosyl-(1–2)-galactosylceramide (GGC) were synthesized using established methods.

##### Whole Cell Lysis, Glycosidase Treatment, and Western Blotting

The indicated cell lines were plated in 150-mm plates at either 2.5 million cells/plate on day −2 or 5 million cells/plate on day −1. On day 0, cells were detached and counted, and equal numbers of cells/sample were solubilized on ice in 1% Triton/Tris-buffered saline (TBS), pH 7.4, supplemented with a protease inhibitor mixture (Complete, EDTA-free, Roche Applied Science). The lysate was spun at 20,000 × *g* on a tabletop centrifuge at 4 °C for 10 min to prepare post-nuclear supernatant. Protein concentration was measured using a Bradford assay (Bio-Rad). Equal amounts of protein, as indicated, were electrophoresed and transferred to polyvinylidene fluoride (PVDF) membranes. Primary antibody dilutions used were: D5, 1:5000; UGT1, 1:1000; GFP, 1:2000. After primary antibody incubation, membranes were probed with horseradish peroxidase coupled secondary antibody (1:5000) or streptavidin (Jackson ImmunoResearch). Detection was done using the Supersignal reagent (Thermo Scientific). For experiments involving EndoH or peptide *N*-glycanase F digestion, 30 μg of denatured lysate was digested overnight with 200 units of enzyme according to the manufacturer's protocol.

##### Metabolic Labeling and Immunoprecipitation

On day −1, mouse embryonic fibroblast transfectants were plated at 5 × 10^6^ cells/plate in 150-mm plates. On day 0, cells were pulse-labeled for 15 min with ^35^S labeling mix (PerkinElmer Life Sciences) at 1 mCi/2.4 × 10^7^ cells. The number of cells to be labeled was calculated such that there would 1.5–2 × 10^6^ cells/lane on the final gels. After labeling, cells were chased in fresh medium with excess unlabeled methionine/cysteine. At the indicated time points, equal aliquots were removed, pelleted, and washed with cold PBS. Cells were then solubilized in 1 ml of 1% digitonin, TBS, pH 7.4, supplemented with a protease inhibitor mixture (Complete, EDTA-free). After preparation of post-nuclear supernatant, lysates were precleared with Protein A beads and normal rabbit serum. Lysates were then immunoprecipitated with antibody CD1d51 (specific for CD1d-β_2_m, 6 μg/sample), D5 (specific for free heavy chain, 3 μg/sample), or both, as indicated in the figure legends. The beads were then stripped by heating to 95 °C in 1% sodium dodecyl sulfate (SDS), TBS, pH 7.4, with 5 mm dithiothreitol. The eluted material was then diluted 10-fold into 1% Triton X-100, TBS with 10 mm iodoacetamide, and the denatured stripped protein was re-immunoprecipitated with D5 antibody (3 μg/sample) and protein A-Sepharose to increase specificity. Immunoprecipitates were then electrophoresed on 12% gels. CD1d-β_2_m and free heavy chain were quantified using ImageQuant 5.2 software (Molecular Dynamics) after exposure of dried gels to phosphorimaging screens. When Endo H digestion was performed, precipitated proteins were eluted using Endo H denaturing buffer and incubated overnight with 100 units of EndoH.

##### Flow Cytometry

0.5 × 10^6^ cells/sample were stained using indicated antibody concentrations as previously described (38) and analyzed using a BD Biosciences FACSCalibur. The data were analyzed and plotted using the FlowJo software (Tree Star Inc, Ashland, OR).

##### NKT Hybridoma Stimulation Assay

CD1d-transduced KO.UGT1− or KO.UGT1+ cells were used as the APCs for these assays. On day −1, cells were plated at 5 × 10^6^ cells/plate in 150-mm tissue culture plates. On day 0, cells were detached and replated in 100λ hybridoma culture medium at the indicated concentrations (from 30,000 to 100,000 cells/well) in 96-well plates. After allowing 5 h for cells to attach, indicated hybridomas were added at 100,000 cells/well. After an overnight co-culture, the IL-2 concentration in the supernatant was measured using the Opti-ELISA kit from BD Biosciences according to the manufacturer's instructions. For experiments involving glycolipid loading, the glycolipid was dispersed from a DMSO stock into culture medium at the indicated concentration by sonication at 37 °C for 15 min. The glycolipid was pulsed for 5 h during the APC reattachment period. APCs were then thoroughly washed with medium five times to remove excess lipid, and hybridoma cells were then added in the absence of lipid for overnight co-culture.

## RESULTS

### 

#### 

##### CD1d-β_2_m Heterodimers Form More Rapidly in the Absence of UGT1

**FIGURE 1.**
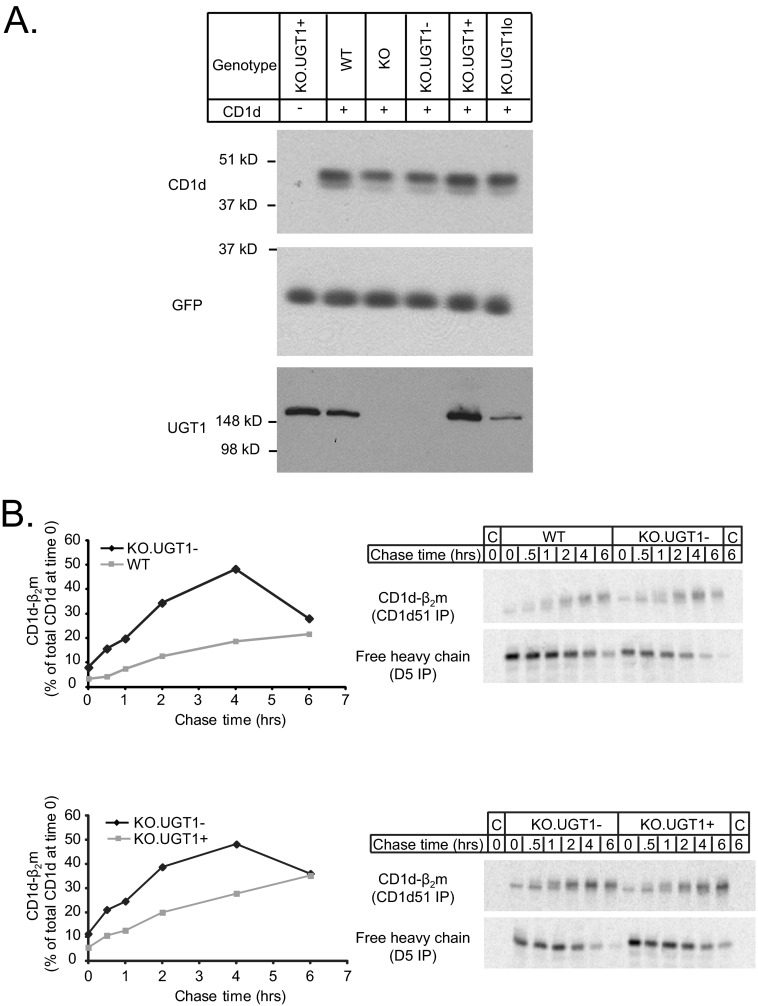
*A*, there is a trend toward lower steady-state CD1d levels in UGT1-deficient cells. 6 × 10^6^ cells/cell line were solubilized in 1% Triton, TBS to prepare whole cell lysates. 12 μg of protein was separated by SDS-PAGE (12% acrylamide), transferred to a PVDF membrane, and probed with monoclonal antibodies for CD1d (D5) and GFP (as a loading control). 65 μg of each lysate was also electrophoresed on an 8% gel, transferred, and blotted with a polyclonal antibody for UGT1. *B*, shown is faster assembly of CD1d-β_2_m complexes in the absence of UGT1. WT, KO.UGT1−, or KO.UGT1+ cells were labeled for 15 min with [^35^S]methionine/cysteine and then chased in cold medium for the indicated amounts of time. Cells were then solubilized in 1% digitonin, TBS. Lysates were divided in half and immunoprecipitated (*IP*) with either antibody CD1d51 (specific for CD1d-β_2_m) or antibody D5 (specific for free heavy chain). To maximize specificity, we used a sequential immunoprecipitation protocol (see “Experimental Procedures”). To assess the rate at which newly synthesized CD1d heavy chains form CD1d-β_2_m complexes, we calculated the amount of heavy chain precipitated by CD1d51 (*i.e.* CD1d-β_2_m) at each time point as a percent of the total heavy chain at time 0 (CD1d51 signal+D5 signal). To ensure specificity we also performed the experiment in parallel on cells untransfected with CD1d (*first* and *last lane* on each gel, labeled *C* for control).

To investigate the role of UGT1-mediated quality control in CD1d assembly, we used wild-type (WT) and UGT1-deficient (KO) mouse embryonic fibroblasts (33). As a more stringent test of the specific effects of UGT1 deficiency, we compared KO cells with cells in which UGT1 expression had been reconstituted. To generate the reconstituted cells, KO cells were transduced with the bicistronic retroviral vector MSCV-UGT1-IRES-Thy1.1. The “empty” vector MSCV-IRES-Thy1.1 was used as a negative control. Using Thy1.1 as a surrogate marker for fluorescence-assisted cell sorting (FACS), we separated out a cell line expressing levels of UGT1 close to those seen in WT cells. These cells are henceforth referred to as KO.UGT1+ cells, and cells transduced with the empty vector are referred to as KO.UGT1− cells. All cell lines were also transduced with constructs expressing human CD1d (MSCV-CD1d-IRES-GFP). Using GFP as a transduction marker, we used FACS sorting to isolate pure populations of transduced cells with similar CD1d expression levels. Fig. 1*A* shows the expression of CD1d, GFP, and UGT1 in WT, UGT1-deficient (KO and KO.UGT1−), and reconstituted cell lines (KO.UGT1+ and KO.UGT1lo). KO.UGT1+ and KO.UGT1lo are cell lines expressing different levels of UGT1, with KO.UGT1+ most closely approximating wild-type levels. We observed a trend toward lower steady-state levels of CD1d in the UGT1-deficient cells despite equivalent levels of GFP expression, suggesting a possible defect in CD1d folding and/or assembly.

To explore this possibility further, we examined the early steps in CD1d maturation. Previous work has demonstrated that lectin-chaperone mediated CD1d heavy chain folding and disulfide bond formation precedes β_2_m association of newly synthesized CD1d (16). Indeed, in CRT-deficient cells, the rate of assembly of CD1d-β_2_m heterodimers was higher (24). We reasoned that if UGT1 monitors the formation of mature CD1d complexes, accelerated formation of heterodimers may also be expected in UGT1 null cells. To test this, WT and KO.UGT1− cells were pulsed with [^35^S]methionine/cysteine for 15 min and chased up to 6 h. At various time points, cells were solubilized in 1% digitonin to maintain CD1d-β_2_m association (38). The lysates were then divided and immunoprecipitated with either antibody CD1d51 (specific for CD1d-β_2_m heterodimers) or D5 (specific for free heavy chains) (37). To ensure specificity of the immunoprecipitation, a sequential immunoprecipitation protocol was used (as detailed under “Experimental Procedures”), and lysates of untransfected cells were used as controls. As seen in Fig. 1*B*, most of the labeled CD1d was in the D5-reactive (free heavy chain) form at time 0 in both cell lines. Over time, this population converted to CD1d51-reactive heterodimers. To assess the rate of dimerization, at each time point we calculated the amount of CD1d-β_2_m heterodimer as a percentage of the total CD1d signal (D5+CD1d51) at time 0. In general, at every early time point after the pulse, a higher fraction of newly synthesized CD1d was found in the CD1d51-reactive form in KO.UGT1− cells compared with WT cells. Thus, as in the CRT-deficient cells, CD1d-β_2_m heterodimer assembly occurs at a faster rate in the absence of UGT1. Interestingly, in UGT1 null cells, the amount of heterodimerized CD1d reached a maximum at 4 h and diminished significantly by 6 h. In contrast, in UGT1-sufficient cells, the fraction of heterodimerized CD1d rose steadily throughout the course of the experiment and then either reached a plateau or showed a slow decay (Fig. 1*B*; see Fig. 5*C*). This suggests that at least a subset of the CD1d-β_2_m heterodimers formed in UGT1-deficient cells is shorter-lived and potentially unstable. We also compared KO.UGT1− cells to KO.UGT1+ cells and observed similar results (Fig. 1*B* and supplemental Fig. S1).

##### CD1d-β_2_m Heterodimers in UGT1 Null Cells Are Competent for Rapid ER Export

**FIGURE 2.**
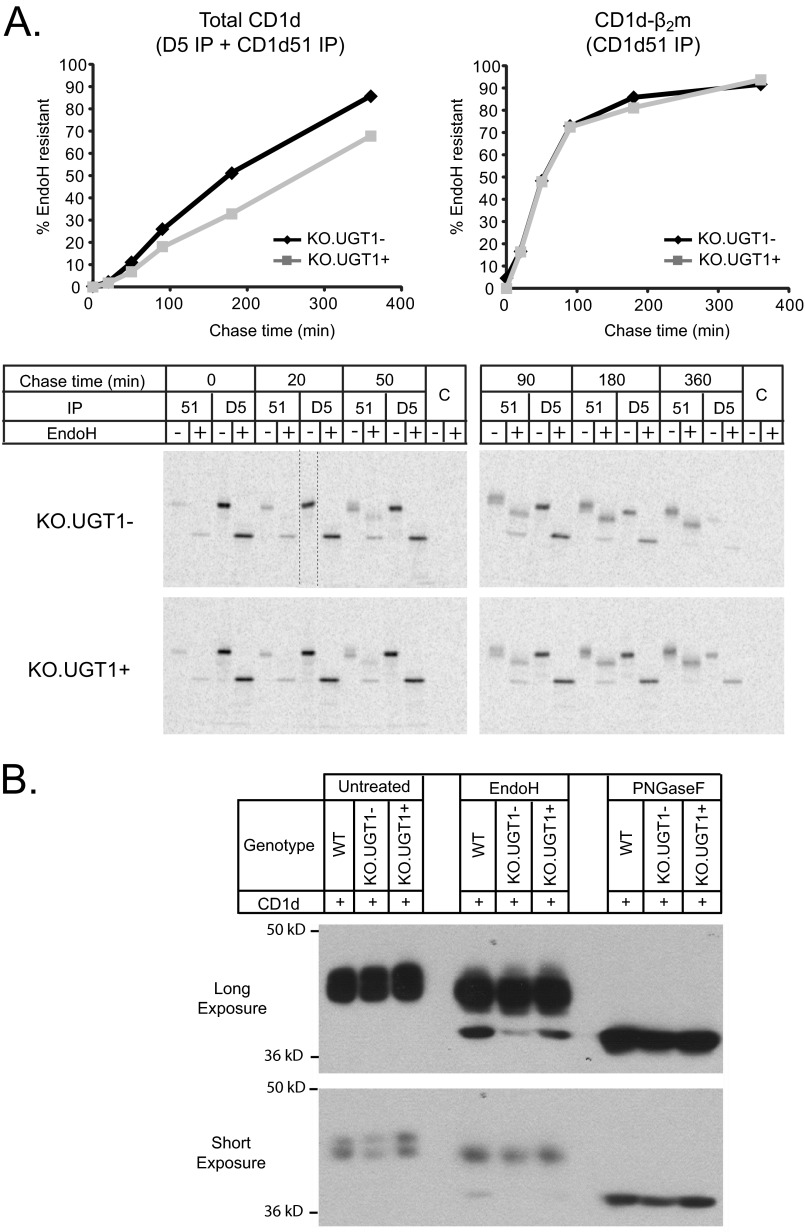
*A*, CD1d-β_2_m complexes in UGT1-deficient cells are competent for rapid ER export. KO.UGT1− or KO.UGT1+ cells were pulse-labeled, chased, and subjected to sequential immunoprecipitation as in Fig. 1*B*. Immunoprecipitates (*IP*) were eluted by boiling, treated either with or without EndoH, and then quantified after electrophoresis (see “Experimental Procedures”). We plotted the percent of total CD1d (CD1d51-reactive+D5-reactive) that is EndoH-resistant at each time point (*left panel*) and the percent of CD1d-β_2_m (CD1d51-reactive) that is EndoH-resistant (*right panel*). There is a faster rate of acquisition of EndoH resistance in UGT1 null cells when examined as a percent of all CD1d molecules, but the rate is identical when only heterodimerized CD1d is examined. This indicates that the faster acquisition of EndoH resistance in UGT1-deficient cells is explained by faster heterodimerization kinetics and that the faster-formed complexes in null cells are competent for rapid ER export. The *lane bordered by dashed lines on the top left gel* was run as the last lane on the same gel and has, therefore, been cut and pasted into the appropriate place for clarity using Adobe Photoshop. *B*, less CD1d is ER-retained in UGT1-deficient cells at steady state. WT, KO.UGT1−, and KO.UGT1+ cells were solubilized in 1% Triton, TBS, and equal amounts of protein were subjected to EndoH or peptide *N*-glycanase F digestion (see “Experimental Procedures”). 8 μg of protein/sample was separated by SDS-PAGE and blotted with D5 (CD1d heavy chain). The level of EndoH-sensitive CD1d is much lower in UGT1-deficient cells, whereas the level of EndoH-resistant protein is similar to WT or reconstituted cells. Peptide *N*-glycanase F (*PNGaseF*) serves as a control to indicate that the lower band in the EndoH lanes is indeed completely deglycosylated and, therefore, EndoH-sensitive. Mature CD1d remains partially EndoH-sensitive as glycan 2, at the interface of CD1d and β_2_m, is protected from Golgi-associated sugar modifications (38). All cells were treated with 100 μm chloroquine overnight to remove any confounding issues caused by differential lysosomal degradation.

Having determined that CD1d-β_2_m heterodimer formation is accelerated in UGT1-deficient cells, we next assessed whether these complexes are competent to exit the ER. KO.UGT1− or KO.UGT1+ cells were subjected to pulse-chase analysis involving digitonin extraction and immunoprecipitation with CD1d51 or D5 as above. Immunoprecipitates of either free heavy chain or CD1d-β_2_m dimer were digested with EndoH to assess their ER-export status (Fig. 2*A*). As before, we observed that the formation of CD1d-β_2_m heterodimers was accelerated in UGT1-deficient cells (supplemental Fig. S2). The fraction of total CD1d that was EndoH-resistant at each time point was calculated. We observed that the rate of acquisition of EndoH resistance was faster in UGT1-deficient cells. In addition, a higher fraction of newly synthesized CD1d acquired EndoH resistance (Fig. 2*A*, *left panel*). However, when we specifically examined β_2_m-associated CD1d free heavy chains, the rate of acquisition of EndoH resistance was identical in the absence and presence of UGT1. These observations indicate that the increased rate of EndoH acquisition is accounted for by faster heterodimerization with β_2_m, whereas the heterodimers exit the ER at the same rate once formed. We did not observe EndoH-resistant free heavy chain in either the UGT1-deficient or -sufficient cells, indicating that free heavy chains failed to leave the ER. This also confirms that no free heavy chains were derived from digitonin-induced dissociation of CD1d-β_2_m complexes that had left the ER. Overall, the results indicate that the faster assembled CD1d-β_2_m heterodimers in UGT1-deficient cells are competent for ER export.

We also assessed how these kinetic differences in CD1d assembly and trafficking affect the steady-state distribution of cellular CD1d. We used EndoH digestion to assess the relative amounts of ER and post-ER CD1d in unlabeled WT, KO.UGT1−, and KO.UGT1+ cells (Fig. 2*B*). Much reduced levels of ER-retained CD1d were found in UGT1-deficient cells compared with WT or reconstituted KO.UGT1+ cells, whereas the level of post-ER CD1d was similar. This is consistent with the faster formation of export-competent CD1d-β_2_m heterodimers observed in our kinetic assays.

##### Steady-state Surface Expression of CD1d-β_2_m Heterodimers Is Unaffected in UGT1-deficient Cells

**FIGURE 3.**
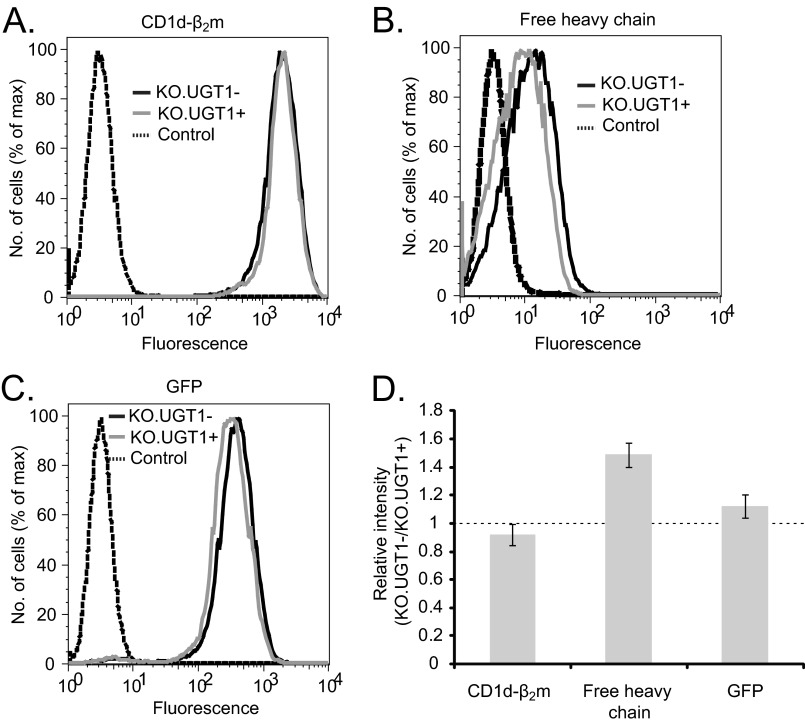
**Equal cell surface levels of CD1d-β_2_m in UGT1-deficient and -sufficient cells at steady state.** Cell surface levels of CD1d-β_2_m (*A*) or free heavy chain (*B*) were examined by flow cytometry in KO.UGT1− cells (*solid black lines*) and KO.UGT1+ cells (*solid gray lines*). Mouse embryonic fibroblast cells untransfected for CD1d were used as controls for the specificity of staining (*dashed black lines*). We used antibody CD1d51 (15 μg/ml) to detect CD1d-β_2_m and antibody D5 (14 μg/ml) to detect CD1d free heavy chain. GFP (*C*) is expressed from an IRES in the retroviral construct after the CD1d open reading frame and, therefore, serves as a transfection control. For *panel D*, we calculated the relative intensity of signal in UGT1-deficient cells compared with reconstituted cells (KO.UGT1− signal/KO.UGT1+ signal). The graph depicts the mean ± S.E. of five independent samples.

iNKT cell recognition is mediated by the subset of CD1d molecules at the cell surface. Given the differences in CD1d assembly and ER exit, we wished to determine whether cell-surface CD1d levels are affected by UGT1 deficiency. We used antibodies CD1d51 or D5 to measure cell surface levels of CD1d-β_2_m heterodimers or free heavy chain, respectively, by flow cytometry (Fig. 3, *A–C*). We observed that the surface expression of CD1d-β_2_m heterodimers was identical between KO.UGT1− and KO.UGT1+ cells. KO.UGT1− cells had slightly but consistently higher levels of D5-reactive free heavy chain on the cell surface (averaged over multiple experiments, the level of surface free heavy chain was 1.4 times greater in UGT1-deficient cells; Fig. 3*D*). This could occur either because of increased release of free heavy chains from the ER or easier dissociation of unstable CD1d heterodimers after export. Regardless, it is important to note that cell surface D5 staining is extremely low in either cell line, consistent with the lack of detectable free Endo H-resistant CD1d heavy chains in the pulse-chase experiments depicted in Fig. 2*A*. Overall, these results indicate that there is minimal change in surface CD1d expression in UGT1-deficient cells despite faster complex assembly and ER egress. This is consistent with the shorter half-life of CD1d-β_2_m heterodimers observed in our pulse-chase experiments (Fig. 1*B*) and raised the possibility that these complexes may be qualitatively different.

##### Altered Antigen Presentation by CD1d in the Absence of UGT1

**FIGURE 4.**
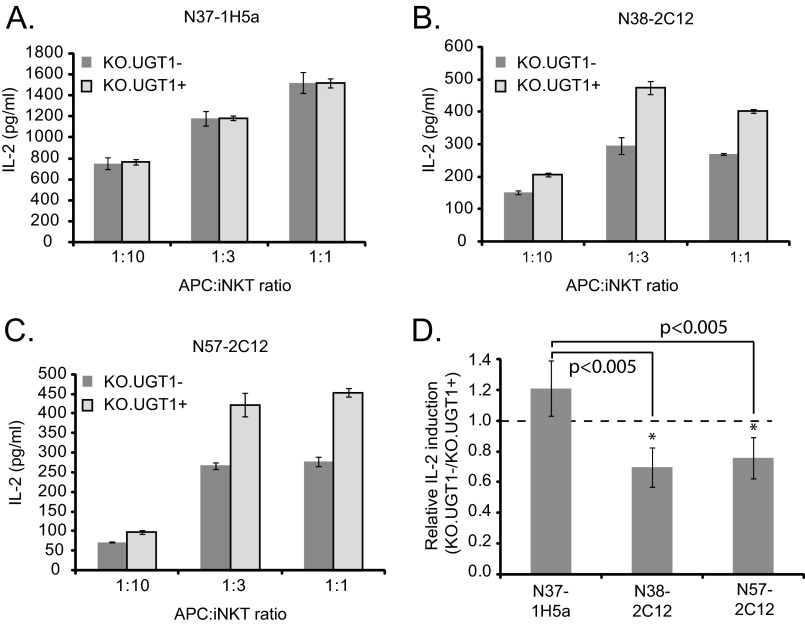
**Altered stimulation of a subset of self-reactive iNKT cell hybridomas by UGT1-deficient cells.** *A–C*, KO.UGT1− cells or KO.UGT1+ cells were co-cultured overnight with three self-reactive iNKT cell hybridomas that have different TCRβ sequences (N37-1H5a, Vβ8.2-Jβ2.6; N38-2C12, Vβ8.2-Jβ2.5; N57-2C12, Vβ14-Jβ1.2) (34, 35). Three different APC:iNKT ratios were tested by altering the number of APCs while keeping the number of iNKT cells constant. IL-2 levels in the supernatant were measured by ELISA. UGT1-deficient cells elicit significantly lower IL-2 production from hybridomas N38-2C12 and N57-2C12 but are unaffected in their ability to stimulate the N37-1H5a hybridoma. These differences were consistent for all APC:iNKT ratios, *D*, in four additional independent experiments, we calculated the relative stimulation of the same three iNKT hybridomas by UGT1-deficient cells compared with reconstituted cells (ratio of IL-2 induction by KO.UGT1− cells/IL-2 induction by KO.UGT1+ cells). We plotted the mean ± S.D. of this ratio across the four experiments. The APC:iNKT ratio for these experiments was 1:3. The differences in stimulation profile of the three hybridomas were consistent across the multiple experiments. (*, paired two-tailed *t* test for comparison to N37-1H5a, *p* value <0.005).

Having ruled out a quantitative difference in cell-surface CD1d, we investigated whether there was a functional difference (*i.e.* a difference in antigenicity) in CD1d-β_2_m complexes between UGT1-deficient and -sufficient cells. We tested this by comparing the ability of KO.UGT1− and KO.UGT1+ cells to stimulate a panel of three auto-reactive iNKT cell hybridomas previously shown to carry different TCR β chains and to have different reactivities to various CD1d-lipid combinations (34, 35). We co-cultured KO.UGT1− or KO.UGT1+ cells overnight with the hybridomas at varying APC:iNKT ratios and measured IL-2 levels in the supernatant to detect iNKT cell activation (Fig. 4). KO.UGT1− and KO.UGT1+ cells activated hybridoma N37-1H5a equally well, in keeping with their similar levels of CD1d surface expression. However, two other hybridomas (N38-2C12 and N57-2C12) demonstrated significantly reduced activation with KO.UGT1− cells compared with KO.UGT1+ cells. These trends were consistent over all APC:iNKT ratios tested and over multiple experiments (Fig. 4*D*). The altered activation of these two hybridomas, coupled with identical cell surface CD1d-β_2_m levels in the UGT1-deficient and -sufficient cells, strongly indicate that CD1d-β_2_m complexes generated in the absence of UGT1 are qualitatively different.

**FIGURE 5.**
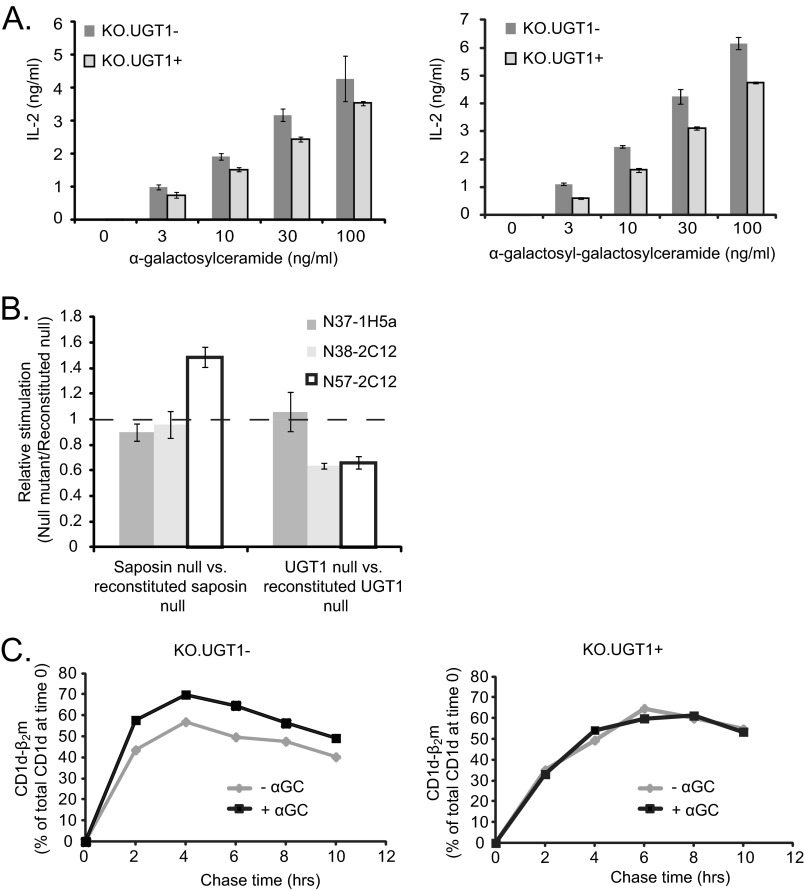
*A*, exogenous lipid antigens are presented better by UGT1-deficient cells. KO.UGT1− or KO.UGT1+ cells were pulsed with the indicated concentrations of two related glycolipid antigens for 4 h, washed extensively, and then co-cultured overnight with the iNKT cell hybridoma DN32.D3. GGC is a variant of αGC that requires lysosomal processing before it can be recognized by iNKT cells. All co-cultures were done at an APC:iNKT ratio of 1:3. *B*, iNKT hybridoma stimulation profile for saposin-deficient cells is distinct from that for UGT1-deficient cells. We performed a co-culture experiment to assess the stimulatory capacity of saposin null cells (PSK02.CD1d or F89.39.CD1d) (40) relative to their reconstituted counterparts (PSK02.CD1d.prosaposin or F89.39.CD1d.PS). This was done in parallel with a co-culture experiment comparing KO.UGT1− and KO.UGT1+ cells. The three self-reactive iNKT cell hybridomas described in Fig. 4 were used as effector cells in the assay. We calculated the relative stimulation of each deficient cell line to its reconstituted counterpart by calculating the ratio of IL-2 in the supernatant after overnight co-culture. The two hybridomas whose stimulation was impaired when using UGT1-deficient cell lines (N38-2C12 and N57-2C12) did not show this defect when stimulated by saposin-deficient cells. *C*, a better presentation of αGC by UGT1-deficient cells correlates with increased abundance of CD1d-β_2_m complexes. KO.UGT1− or KO.UGT1+ cells were incubated overnight either with or without 100 ng/ml αGC. Cells were then pulsed with [^35^S]methionine/cysteine, chased again with or without αGC, and solubilized in 1% digitonin, TBS. After immunoprecipitation of lysates with the appropriate antibodies (see “Experimental Procedures” and Fig. 1*B*), we calculated the percent of initially labeled free heavy chain present in CD1d-β_2_m at each time point (see supplemental Fig. S3 for SDS-PAGE images). In UGT1-deficient cells, αGC treatment caused a greater percentage of initially labeled heavy chain to accumulate in CD1d-β_2_m complexes during the course of the experiment. This difference was not observed in KO.UGT1+ cells.

Having tested the presentation of endogenous antigens, we also tested the ability of CD1d complexes to load and present exogenous antigens in UGT1-deficient cells. We employed two commonly used model antigens: αGC, which is capable of loading onto CD1d either at the cell surface or in the endocytic system (39, 40), and GGC, which requires lysosomal processing by α-galactosidase for activity (41). After pulsing KO.UGT1− or KO.UGT1+ cells with antigen, we performed co-culture experiments similar to the experiments described above. For detection, we chose the hybridoma DN32.D3 because it has minimal auto-reactivity and is strongly activated by αGC (36, 42). Interestingly, for both antigens, we observed that UGT1-deficient cells activate the DN32.D3 hybridoma better than UGT1-sufficient cells (Fig. 5*A*).

We considered the possibility that the antigen presentation phenotype may be caused by effects on accessory molecules required for lipid transfer onto CD1d. In particular, prosaposin (the precursor for lysosomal saposins) is known to be a UGT1 substrate (43). Given that saposins are required for lysosomal loading and presentation of exogenous antigens (40, 44), better presentation of αGC and GGC in UGT1-deficient cells argued against this possibility. However, to further examine this question, we compared the auto-reactive hybridoma stimulation profile of saposin-deficient cells and UGT1-deficient cells (Fig. 5*B*). The defect in stimulation of the N38-2C12 and N57-2C12 hybridomas seen in UGT1-deficient cells was clearly not seen in saposin-deficient cells.

##### Strong Antigens Stabilize CD1d-β_2_m Complexes in UGT1-deficient Cells

Our kinetic data (Figs. 1*B* and 2) suggested that CD1d-β_2_m complexes in UGT1-deficient cells are shorter-lived and possibly unstable. Therefore, a potential explanation for the superior presentation of exogenous antigens by UGT1-deficient cells is that these unstable CD1d-β_2_m heterodimers can be stabilized by binding the antigen. This is analogous to classic observations that peptide antigens can bind more readily to MHC class I molecules and stimulate CD8-positive T cells better in cells lacking the Transporter associated with Antigen Processing (TAP) or other components required for proper peptide loading (45–47). We tested this hypothesis using pulse-chase assays to monitor the accumulation of CD1d-β_2_m complexes in cells cultured either in the absence or presence of αGC (Fig. 5*C* and supplemental Fig. S3). KO.UGT1− and KO.UGT1+ cells were incubated overnight either with or without αGC, labeled, and then chased either with or without αGC. At the indicated times, cells were solubilized in digitonin and CD1d-β_2_m complexes immunoprecipitated as described previously. We quantitated the accumulation of CD1d-β_2_m heterodimers by measuring the percentage of CD1d heavy chains present at time 0 that accumulated in CD1d-β_2_m complexes at various chase times. As expected, in KO.UGT1− cells, we observed a higher percentage of heterodimerized CD1d in the presence of αGC. This difference was not seen in the KO.UGT1+ cells that were tested in parallel, suggesting that “rescuable” unstable CD1d-β_2_m complexes are not present in these cells.

**FIGURE 6.**
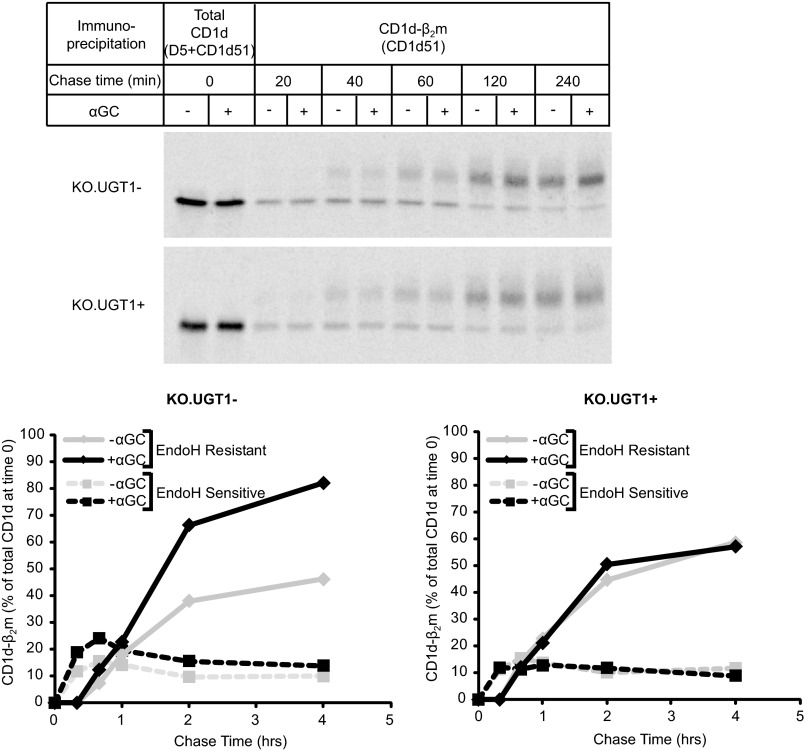
**The effect of αGC on CD1d-β_2_m complex abundance occurs mainly in a post-ER compartment.** KO.UGT1− or KO.UGT1+ cells were incubated overnight either with or without 100 ng/ml αGC. Cells were then pulsed with [^35^S]methionine/cysteine, chased again with or without αGC, and solubilized in 1% digitonin, TBS. After immunoprecipitation with the indicated antibodies using a sequential immunoprecipitation protocol (“Experimental Procedures”), eluates were digested with EndoH. At each time point we calculated the amount of CD1d-β_2_m heterodimer in the EndoH-sensitive or -resistant form in the presence or absence of αGC, expressed as a percent of labeled CD1d at time 0. The αGC-dependent increase in abundance of CD1d-β_2_m complexes in UGT1-deficient cells was largely restricted to the EndoH-resistant fraction. No increase was observed in KO.UGT1+ cells.

We next assessed whether the rescue phenomenon described above affects newly formed CD1d-β_2_m heterodimers in the ER or the post-ER population of CD1d-β_2_m complexes. Our data up to this point indicated that degradation/disassembly of shorter-lived CD1d-β_2_m complexes in UGT1-deficient cells occurs after ER egress. Therefore, if αGC rescue occurs by preventing this process, the rescue effect should be seen largely in the post-ER fraction of CD1d-β_2_m. We tested this prediction in a pulse-chase experiment in which CD1d-β_2_m complexes were immunoprecipitated at various chase points as before, and immunoprecipitates were subjected to EndoH digestion. At each time point, we quantified the amount of CD1d-β_2_m heterodimer in the EndoH-sensitive or -resistant form in the presence or absence of αGC, expressed as a percent of labeled CD1d at time 0 (Fig. 6). As predicted, the stabilizing effect of αGC was restricted almost completely to the EndoH-resistant fraction. Also, as expected, we did not observe stabilization in either the EndoH-sensitive or -resistant fractions in KO.UGT1+ cells.

## DISCUSSION

In this study we have reported our analysis of CD1d assembly and function in cells lacking the enzyme UGT1, a key component of the ER glycoprotein quality control machinery. The major observations reported are the following. 1) In UGT1-deficient cells, CD1d-β_2_m complexes are formed at a faster rate and in a higher proportion (Fig. 1*B*). 2) These faster-formed CD1d-β_2_m complexes are competent for rapid ER export (Fig. 2). 3) The increased accumulation of CD1d-β_2_m heterodimers is transient (Fig. 1*B*, compare the 6-h time point to earlier ones). 4) These complexes can be stabilized by providing a high affinity lipid antigen (Figs. 5*C* and 6). 5) CD1d-β_2_m complexes in UGT1-deficient cells have altered antigenicity (Fig. 4) that is not due to a global defect in CD1d function (compare hybridoma N37-1H5a to the others in Fig. 4 and also see Fig. 5*A* where UGT1-deficient cells actually present exogenous antigens better).

The data allow us to conclude that UGT1-mediated and lectin-chaperone-dependent quality control affects CD1d assembly and maturation. Importantly, we can also conclude that the quality control process has a significant effect on CD1d function. The most likely explanation for our findings is that, in the absence of UGT1-mediated quality control, suboptimal CD1d-β_2_m heterodimers escape the ER. At least a subset of these complexes is short-lived. This may either be due to lysosomal degradation after endocytosis, dissociation at the cell surface, or a combination of both phenomena. In support of the latter, we did observe slightly higher levels of cell-surface free heavy chain in UGT1 null cells (Fig. 3). Furthermore, Odyniec *et al.* (48) have recently shown that cell surface CD1d has an inherent instability that is likely to be dependent on the loaded lipid. These observations suggest that cell surface dissociation may contribute at least partially to the relative instability of CD1d-β_2_m complexes in UGT1 null cells. Regardless of the precise mechanism involved, the end result of the combination of greater ER export and decreased stability is an equivalent or near-equivalent level of total CD1d on the cell surface of UGT1-deficient and -sufficient cells. An additional point of support for this assertion is the fact that the N37-1H5a hybridoma is stimulated equally well by UGT1-deficient and sufficient cells (Fig. 4*A*).

Against the backdrop of equal CD1d levels at the cell surface, we clearly observe a difference in antigenicity that is specific to only a subset of iNKT cell hybridomas. This observation suggests that the absence of UGT1-mediated quality control affects the identity of CD1d-loaded antigens. Our observation that provision of αGC is able to rescue CD1d-β_2_m complexes greatly strengthens this possibility. The fact that the exogenous antigenic lipids are presented better by UGT1-deficient cells argues against an inherent structural defect in the CD1d-β_2_m heterodimers. Such antigen-dependent stabilization has been described well for MHC class I (45–47) and is consistent with the ligand-dependent stabilization of cell-surface CD1d observed by Odyniec *et al.* (48). Finally, we observed better presentation even with GGC, a lipid that requires lysosomal processing for recognition. This indicates that the rescued complexes are stable even after lysosomal trafficking, again arguing against an inherent instability in the CD1d heavy chain in the absence of UGT1.

We considered the possibility that the observed differences in CD1d assembly and/or antigenicity may be caused by UGT1 dependence of an accessory molecule involved in CD1d assembly or loading. The fact that exogenous antigens rescue both stability as well as antigenicity of CD1d strongly implies that the assembly phenotype and the antigenicity differences are causally related. Therefore, any candidate accessory molecule would have to be one that has the ability to load/unload CD1d-bound lipids. It is known that prosaposin is a substrate for UGT1. We believe it is very unlikely that our findings are explained by an indirect effect on saposin for three reasons; 1) we tested and found completely different self-antigenicity profiles for UGT1 null and saposin null cells (Fig. 5*B*); 2) unlike UGT1 null cells, saposin null cells have lower responses to αGC and GGC (40, 44); 3) although secreted prosaposin is greatly reduced in UGT1 null cells, levels of processed saposins appear to be relatively preserved (43). Additionally, deficiencies of all other known or putative lysosomal accessory molecules involved in CD1d loading lead to diminished presentation of αGC (1), a phenotype opposite to the one we observe in UGT1 null cells. Deficiencies in lysosomal lipid transfer proteins would also not explain the premature hetero-dimerization and ER export we observe in UGT1 deficiency. The only ER-resident accessory molecule known to be involved in CD1d assembly/loading is the lipid transfer protein (MTP). Again, the phenotype described for MTP deficiency (lower cell surface CD1d levels and lower αGC responses) is distinct from the phenotype we observe in UGT1 null cells (17, 49). Although it is formally possible that the phenotypes we observe are caused by misfolding or malfunction of an unknown ER-resident accessory molecule involved in shaping the CD1d lipid repertoire, none is known that could explain these phenotypes. Therefore, we believe that the data indicate a direct role for UGT1 in the quality control of CD1d assembly.

Previous work has demonstrated that human CD1d has four *N*-linked glycans (38), and murine CD1d has five glycans (50). The presence of one or more glycans is essential for surface expression and function of CD1d in both systems. Interestingly, in human CD1d, all four *N*-linked glycans are present in the α1 and α2 domains, effectively surrounding the antigen-binding site. It is tempting to speculate, then, that subtle changes in the conformation of the antigen binding groove caused by the presence or absence of an antigen (or the type of antigen) may be detected by UGT1. In fact, the structure of empty CD1d reported by Koch *et al.* (14) does reveal a widening of the antigen binding groove when compared with the lipid-loaded molecule. Such a change would expose the hydrophobic interior of the CD1d lipid binding pocket, a surface that may be suited for detection by UGT1. In fact, the glycan at Asn-42 is positioned at the interface of the CD1d heavy chain and β_2_m and affects the rate of association of the two molecules (38). It is possible, therefore, that glucosylation of this particular glycan by UGT1 causes reassociation with CRT, prolonging ER retention and facilitating proper assembly. This is an attractive explanation but because of the complexity of the effects of removal of each of the individual glycans on CD1d assembly and cell surface expression (38), it is not readily amenable to testing.

Our findings are consistent with and extend previous observations on the assembly and lipid loading of CD1d in the ER. In cells lacking CRT, we have seen a similar phenotype of early heterodimerization and export (24). In contrast to our findings here, in CRT null cells, we observed significantly higher levels of cell surface CD1d-β_2_m. Also, the level of cell surface free heavy chain was significantly higher than that seen here. CRT is involved in the initial folding of CD1d in addition to any role it may play in UGT1-mediated quality control (16). Therefore, it is not surprising that we observed a stronger effect on cell surface CD1d levels in CRT null cells. In addition to core components of the ER quality control machinery, another ER-resident molecule intimately involved with CD1d assembly is MTP (17). MTP is capable of transferring phospholipids onto purified CD1d (19). In the absence of MTP, presentation of both endogenous as well as exogenous antigens is affected, and cell surface levels of CD1d are reduced (17–19, 49). At least in some cell types, this may be due to ER retention of CD1d (17). UGT1-mediated quality control could be responsible for this phenomenon; failure to appropriately load lipids onto newly synthesized CD1d in MTP-deficient cells may lead to UGT1-mediated reglucosylation and thereby result in ER retention of CD1d. Our results regarding the presentation of exogenous glycolipid antigens are opposite to those observed in MTP null cells. This may be because there is a difference between empty CD1d molecules (as would be expected in the absence of MTP) and suboptimally loaded CD1d (as might be seen in the absence of UGT1). Alternatively, MTP has been shown to have a distal effect on the lysosomal lipid loading of CD1d (49), which might account for the difference.

In summary, our data suggest a model wherein UGT1 controls the quality of newly synthesized CD1d complexes. This may be achieved by monitoring of fine structural characteristics in the region of the antigen binding groove. The quality control process influences the antigenicity of CD1d, likely by optimizing the lipid repertoire of newly synthesized CD1d. The pathophysiological relevance of this process is beyond the scope of this study. There are, however, a number of interesting possibilities. To counter the inherent instability of CD1d on the cell surface (48), quality control in the ER may ensure that exported complexes are loaded with lipids capable of maintaining the structural integrity of cell-surface CD1d. More intriguingly, it is possible that the quality control process ensures the presentation of low abundance but high affinity antigens that are transiently present in the ER, such as lysophospholipids (23) or mono-glycosylated ceramides (51). A final consideration is the effect of cellular stress states (such as the unfolded protein response) on the quality control process. It is possible that competition for UGT1 from other substrates in such a situation reduces the fidelity of quality control, thereby initiating or aggravating deleterious iNKT cell responses.

## Footnotes

4 The abbreviations used are:

NKT: natural-killer T
iNKT: invariant NKT
TCR: T-cell receptor
β_2_m: β_2_ microglobulin
APC: antigen-presenting cell
ER: endoplasmic reticulum
CRT: calreticulin
UGT1: UDP-glucose glycoprotein glucosyltransferase
MTP: microsomal triglyceride transfer protein
IRES: internal ribosome entry site
EndoH: endoglycosidase H
αGC: α-Galactosylceramide
GGC: α-galactosyl-(1–2)-galactosylceramide.
